# MiR-195 enhances cardiomyogenic differentiation of the proepicardium/septum transversum by Smurf1 and Foxp1 modulation

**DOI:** 10.1038/s41598-020-66325-x

**Published:** 2020-06-09

**Authors:** Angel Dueñas, Almudena Expósito, María del Mar Muñoz, María José de Manuel, Andrea Cámara-Morales, Fabio Serrano-Osorio, Carlos García-Padilla, Francisco Hernández-Torres, Jorge N. Domínguez, Amelia Aránega, Diego Franco

**Affiliations:** Cardiovascular Development Group, Department of Experimental Biology, University of Jaén, Jaén, Spain

**Keywords:** Cell biology, Developmental biology, Molecular biology

## Abstract

Cardiovascular development is a complex developmental process in which multiple cell lineages are involved, namely the deployment of first and second heart fields. Beside the contribution of these cardiogenic fields, extracardiac inputs to the developing heart are provided by the migrating cardiac neural crest cells and the proepicardial derived cells. The proepicardium (PE) is a transitory cauliflower-like structure located between the cardiac and hepatic primordia. The PE is constituted by an internal mesenchymal component surrounded by an external epithelial lining. With development, cells derived from the proepicardium migrate to the neighboring embryonic heart and progressive cover the most external surface, leading to the formation of the embryonic epicardium. Experimental evidence in chicken have nicely demonstrated that epicardial derived cells can distinctly contribute to fibroblasts, endothelial and smooth muscle cells. Surprisingly, isolation of the developing PE anlage and *ex vivo* culturing spontaneously lead to differentiation into beating cardiomyocytes, a process that is enhanced by Bmp but halted by Fgf administration. In this study we provide a comprehensive characterization of the developmental expression profile of multiple microRNAs during epicardial development in chicken. Subsequently, we identified that miR-125, miR-146, miR-195 and miR-223 selectively enhance cardiomyogenesis both in the PE/ST explants as well as in the embryonic epicardium, a Smurf1- and Foxp1-driven process. In addition we identified three novel long non-coding RNAs with enhanced expression in the PE/ST, that are complementary regulated by Bmp and Fgf administration and well as by microRNAs that selectively promote cardiomyogenesis, supporting a pivotal role of these long non coding RNAs in microRNA-mediated cardiomyogenesis of the PE/ST cells.

## Introduction

Cardiovascular development is a complex developmental process in which multiple cell lineages are involved^1^. Soon after gastrulation, bilateral sets of procardiogenic cells align into the embryonic midline configuring a linear cardiac straight tube^2^. These cellular populations constitute the first heart field and will essentially contribute to the future left ventricle^3,4^. Additional cardiogenic progenitor cells emanate from the medial structures in the gastrulating embryo configuring the second heart field and contributing through both cardiac poles to the addition of the right ventricle and outflow at the arterial pole, and the atrioventricular canal and right and left atrial appendages at the venous poles^4–6^. Beside these cardiogenic fields, extracardiac contribution to the developing heart is provided by the proepicardial derived cells^7–13^.

The proepicardium (PE) is a transitory cauliflower-like structure located between the cardiac and hepatic primordia. With development, cells derived from the proepicardium migrate to the developing heart and progressive covers the most external surface^12,13^. Subsequently, the embryonic epicardium is trigger by the underlying myocardium to an epithelial-to-mesenchymal transition (EMT), migrate into the subepicardial space, generating the so-called epicardial derived cells (EPDCs). Thereafter EPCDs migrate into the myocardial layer and differentiate into endothelial, smooth muscle and adventitial cells within the coronary vasculature and fibroblasts and fibrocytes of the cardiac fibroskeleton^10–13^.

Surprisingly, isolation of the developing PE anlage and culturing *ex vivo* spontaneously lead to differentiation of beating cardiomyocytes, a process that is enhanced by Bmp but halted by Fgf administration^14^. These observations lead to postulate that the PE cells have the capacity to become cardiomyocytes but are triggered to adopt a distinct cell fate, opening the possibility of searching for strategies that unlock this halted fate. Importantly, adult epicardium can be triggered to be converted into adult cardiomyocytes by thymosin β4 priming^15^, demonstrating the potentiality of the epicardium to become cardiac muscle and thus opening novel therapeutic opportunities.

microRNAs are a subclass of non-coding RNAs widely and extensively expressed in different tissues during embryonic development, homeostasis and diseases^16^. microRNAs are small RNA molecules of 22–24 nt in length, that contribute to post-transcriptional regulation by base-paired complementary binding to the 3′UTRs of coding RNAs leading to mRNA degradation and/or protein translational blockage^17–20^. Multiple evidences demonstrated the pivotal role of microRNAs during cardiovascular development as evidenced by seminal studies demonstrating that deletion of a single microRNA, i.e. miR-1 and miR-126, respectively, led to embryonic lethality with severe cardiovascular defect^21,22^. Furthermore, manipulation of a discrete number of microRNAs can influence cell fate determination^23,24^.

Evidence of the functional importance of microRNAs in the development of the epicardium was provided by selective inhibition of the key microRNA processing ribonuclease *Dicer* in the epicardial tissue, resulting in thin myocardium and impaired vascular development^25^. These data suggest that microRNA are involved in the cell fate determination process of the embryonic epicardium. However, the functional role of discrete microRNAs in PE remains elusive. In this study we provide a comprehensive characterization of the developmental expression profile of multiple microRNAs during PE and epicardial development in chicken. Subsequently, we identified that *miR-125, miR-146, miR-195* and *miR-223* selectively enhance cardiomyogenesis both in PE/ST explants as well as in embryonic epicardium cultures, a *Smurf1-* and *Foxp1-*driven process. In addition we identified novel three novel long non-coding RNAs with enhanced expression in the PE/ST, that are complementary regulated by Bmp and Fgf administration and well as by microRNAs that selectively promote cardiomyogenesis, supporting a pivotal role of these long non coding RNAs in microRNA-mediated cardiomyogenesis of the PE/ST cells.

## Materials and Methods

### Tissue isolation and culture

Experimental protocols with chicken embryos were performed in agreement with the Spanish law in application of EU Guidelines for animal research. These protocols conformed to the Guide for Care and Use of Laboratory Animals, published by the US National Institutes of Health (NIH publication no. 85–23). Approved consent of the Ethic Committee of the University of Jaen was obtained prior to the initiation of the study. Fertilized eggs from white Leghorn chickens (Granja Santa Isabel, Cordoba, Spain) were incubated at 37.5 °C and 50% humidity for 2–7 days. Embryos were harvested and classified at different developmental stages (HH17, HH24 and HH32) according to Hamburger and Hamilton^26^. Embryos were removed from the egg by cutting the blastocyst margin with iredectomy scissors and placing them into Earle’s balanced salt solution (EBSS) (Gibco). For qPCR analyses, HH17 embryonic hearts and PE/STs, respectively, were isolated, pooled and directly stored at −80 °C until used. HH24 and HH32 hearts were isolated, cultured as described by Ramesh *et al*.^27^, collected, pooled, and stored at −80 °C until used. Epicardial identity was validated by Wt1 and Tbx18 immunohistochemistry, resulting in >80% cells positive for these markers. For *in vitro* explants cultures, chicken HH17 were dissected in Earle’s balanced salt solution (EBSS) (Gibco) and culture into collagen gels as previously described^28^ or, alternatively, cultured in handing drops until collected, pooled and stored at −80 °C until used.

### microRNA and siRNA transfections

HH17 PE explants were cultured on collagen gels or hanging drops for 24 hrs at 37 °C in a cell culture incubator before pre-miRNAs (microRNA precursors) or siRNA transfection, respectively, as previously described^28^. HH24 and HH32 epicardial cells were cultured for 72hrs after the myocardial layer was removed and them transfected. Pre-miRNAs transfections were carried out with Lipofectamine 2000 (Invitrogen), following the manufacturer’s guidelines. Briefly, 85 nM of pre-miRNA were applied to the explants (3–5 explants per well) for 24 hrs. siRNA transfections were also carried out using Lipofectamine 2000 (Invitrogen) as described above. After incubation, explants were either processed for qRT-PCR or immunohistochemical (IHC) analyses. Negative controls, i.e. HH17 explants treated only with Lipofectamine were run in parallel. To perform IHC analyses, the explants were fixed with 1% PFA for 2 hrs at 4 °C, rinsed for three times in PBS during 10 min, and stored in PBS at 4 °C. Each experimental condition was carried out in isolated tissues from at least 30 embryos. In all cases, 3–5 independent biological replicates were analyzed.

### Growth factors and thymosin beta4 administration

PE HH17 explants and HH24 embryonic epicardial primary cultures were treated for 24 h with Bmp2, Bmp4, Fgf2, Fgf8 and thymosin beta 4 (Prospec, East Brunswick, NJ, USA), respectively, as reported by Hinkel *et al*.^29^. Tissue explants were collected and processed according for qPCR and/or immunohistochemistry. Each experimental condition was carried out in isolated tissues from at least 30 embryos. In all cases, 3–5 independent biological replicates were analyzed.

### Cell migration assays

Embryonic epicardial (HH24) primary cell cultures were established similarly as reported by Ramesh *et al*.^27^, but using chicken hearts. Briefly, HH24 embryonic heart were isolated from the developing embryo and the inferior ventricular half was dissected, plated upside down into tissue culture dishes and incubated into DMEM supplemented with glutamine culture media for 48 h. At this stage, emerging epicardial epithelial sheet starting to develop. Transfections with corresponding pre-miRNAs, scrambled and negative controls, respectively, were carried out and immediately placed into the culturing chamber of the time-lapse laser confocal microscope and provided adequate cell tissue culture conditions. Time-lapse analyses was carried for 48 h, with images taken every 30 minutes. Each experimental condition was carried out in isolated tissues from at least 30 embryos. In all cases, 3–5 independent biological replicates were analyzed.

### RNA isolation and qPCR

All qRT-PCR experiments followed MIQE guidelines^30^ and similarly as previously reported^28,31^. Briefly, RNA was extracted and purified by using Trizol reactive (Invitrogen) according to the manufacturer’s instructions. For mRNA expression measurements, 1 μg of total RNA was used for retro-transcription with Maxima First Strand cDNA Synthesis Kit for RT-qPCR (Thermo Scientific). Real time PCR experiments were performed with 1 μL of cDNA, SsoFast EvaGreen mix and corresponding primer sets as described on Supplementary Table 1. For microRNA expression analyses, 20 ng of total RNA was used for retrotranscription with Universal cDNA Synthesis Kit II (Exiqon) and the resulting cDNA was diluted 1/80. Real time PCR experiments were performed with 1 μL of diluted cDNA, ExiLENT SYBR Green master mix (Exiqon) and corresponding primer sets described on Supplementary Table 1. All qPCRs were performed using a CFX384TM thermocycler (Bio-Rad) following the manufacturer’s recommendations. The relative level of expression of each gene was calculated as described by Livak & Schmittgen^32^ using *Gapdh* and *Gusb* as internal control for mRNA expression analyses and *5 S* and *6U* for microRNA expression analyses, respectively. Each PCR reaction was carried out in triplicate and repeated in at least three distinct biological samples to obtain representative means. Heatmaps were obtained using the Multi Experimenter Viewer (version 4.9.0 - Windows 10), of the TM4 software suite^33^. Previously, the normalize function was applied to microRNA expression data, which transform the values using the mean and the standard deviation of the row of the matrix to which the value belongs, using the following formula: Value = [(Value) − Mean(Row)]/[Standard deviation(Row)].

### Immunofluorescence analysis by Confocal scanning laser microscopy

Immunofluorescence analyses were performed as previously reported^28^. Briefly, control and experimental HH17 PE explants and HH24, HH32 epicardial cell cultures were collected after the corresponding treatment, rinsed in PBS for 10 min at room temperature, and fixed with 1% PFA for 2 hrs at 4 °C. After fixation, the samples were rinsed three times (10 min each) in PBS at room temperature and then permeabilized with 1% Triton X-100 in PBS for 30 min at room temperature. To block nonspecific binding sites, PBS containing 5% goat serum and 1% bovine serum albumin (Sigma) was applied to the explants overnight at 4 °C. As primary antibody, a polyclonal goat anti-cardiac troponin I (Hytest) was used, diluted (1:200) in PBS, and applied to each culture overnight at 4 °C. Subsequently, the samples were rinsed three times (for 1 hr each) in PBS to remove excess primary antibody and incubated overnight at 4 °C with Alexa-Fluor 546 anti-goat (1:100; Invitrogen) as secondary antibody. After incubation with the secondary antibody, the explants were rinsed as described above. Finally, the explants and /or epicardial cell cultures, respectively, were incubated with DAPI (1:1,000; Sigma) for 7 min at room temperature and rinsed three times in PBS for 5 min each. Explants were stored in PBS in darkness at 4 °C until analyzed using a Leica TCS SP5 II confocal scanning laser microscope.

### Statistical analyses

For statistical analyses of datasets, unpaired Student’s t-tests were used, as previously reported^28^. Significance levels or P values are stated in each corresponding figure legend. P < 0.05 was considered statistically significant.

## Results

### microRNA profiling during PE/ST and embryonic epicardium development

**Figure 1:**
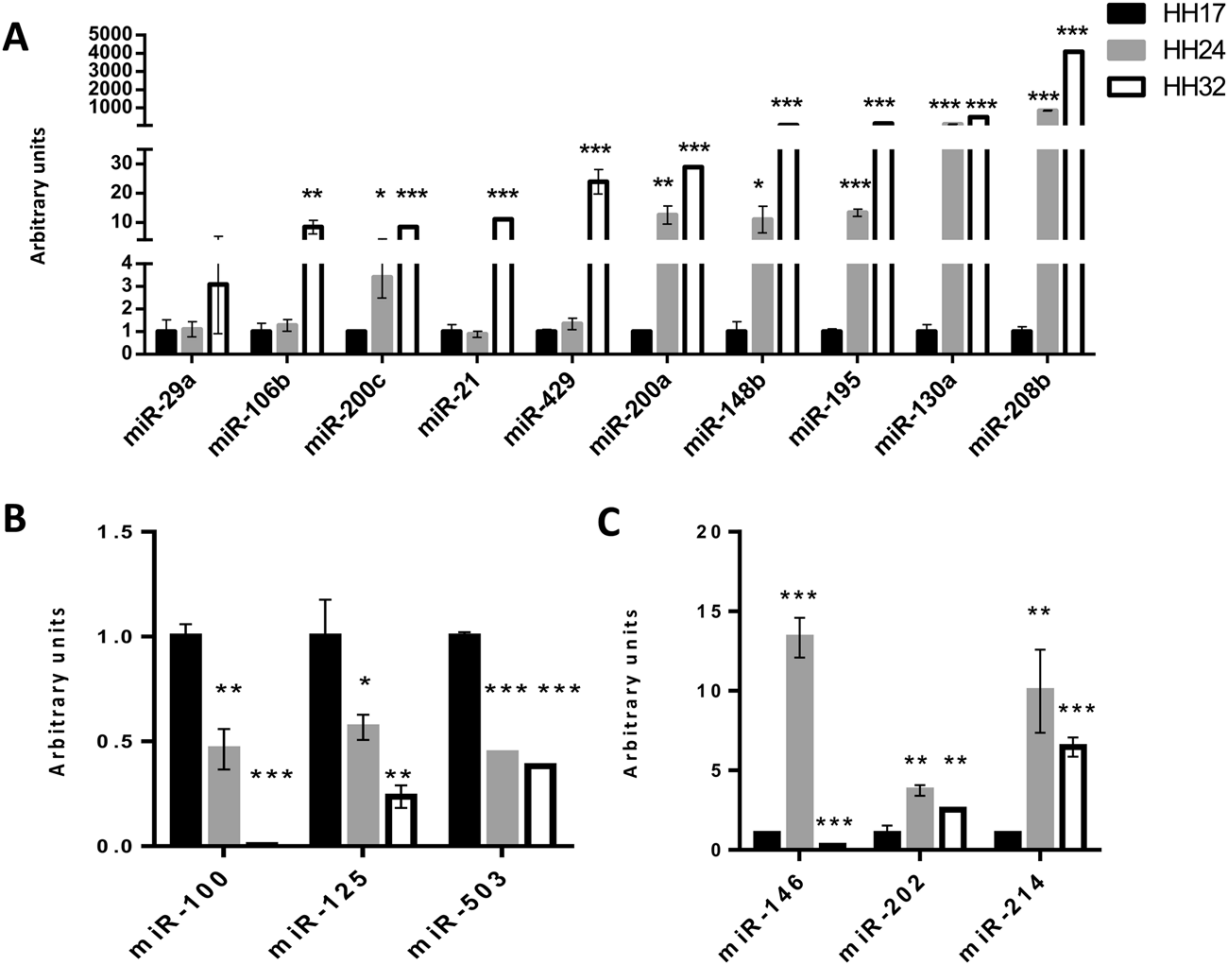
microRNA expression profile during PE and epicardium development. qPCR analyses of the differential expression of microRNAs during PE and epicardium development. Panel A illustrate microRNAs with increasing expression ranging from PE HH17 to embryonic epicardium at HH32. Panel B illustrate microRNAs with decreasing expression ranging from PE HH17 to embryonic epicardium at HH32. Panel C illustrate microRNAs with increased expression ranging from PE HH17 to embryonic epicardium at HH24 but decreasing at H32. HH17 PE were collected from >30 embryos and pooled to performed RNA isolation. Similarly, HH24 and HH32 epicardial cells were collected from >30 ventricular explants. In all cases, three distinct biological replicates of pooled PE/ST, HH24 and HH32 samples were subsequently tested by qPCR.

As previously reported, microRNA function is essential to PE/ST development and contribution to the developing heart^25^. In order to identify differentially expressed microRNAs during epicardial development we have analyzed the expression of distinct microRNAs in three epicardial differentiation stages; HH17 PE/ST explants and HH24 and HH32 cultured embryonic epicardial cells, respectively. 37 microRNAs were selected based on previous results from our laboratory and others that provide evidences of their plausible functional roles in cardiovascular development^34–36^. Among these 37 microRNAs, 16 display differential expression in all distinct epicardial stages (*miR-21, miR-29a, miR-100, miR-106b, miR-125b, miR-130a, miR-146, miR-148b, miR-195, miR-200a, miR-200c, miR-202, miR-208b, miR-214, miR-429, miR-503*) while the remaining 21 (*miR-1, miR-15b, miR-16, miR-22, miR-23a, miR-23b, miR-25, miR-26, miR-27a, miR-31, miR-34a, miR-34c, miR-39, miR-128, miR-130b, miR-185, miR-199a, miR-203a, miR-223, miR-328, miR-448*) display no expression in the PE/embryonic epicardium. Among those displaying differential expression in the developing epicardium, three distinct developmental profiles were observed; a) ten microRNAs were increased over time (*miR-21, miR-29, miR-106b, miR-130a, miR-148, miR-195, miR-200a, miR-200c, miR-208b* and *miR-429*) being barely detectable in the PE/ST explants while significantly increasing as development proceeds (Fig. 1A) b) three microRNAs were decreased over time (*miR-100, miR-125* and *miR-503*), i.e. highly expressed in ST/PE explants while progressively decreasing in HH24 and HH32 stages (Fig. 1B), and c) three microRNAs (*miR-146, miR-202* and *miR-214*) displayed moderate expression levels in HH17 PE/ST, peaking at HH24 and decreasing thereafter at HH32 (Fig. 1C). Overall these data demonstrate a wide dynamic expression profile of distinct microRNAs, suggesting distinct functional roles during epicardial development.

### microRNA mimics modulate cell lineage marker expression in the PE/ST

**Figure 2:**
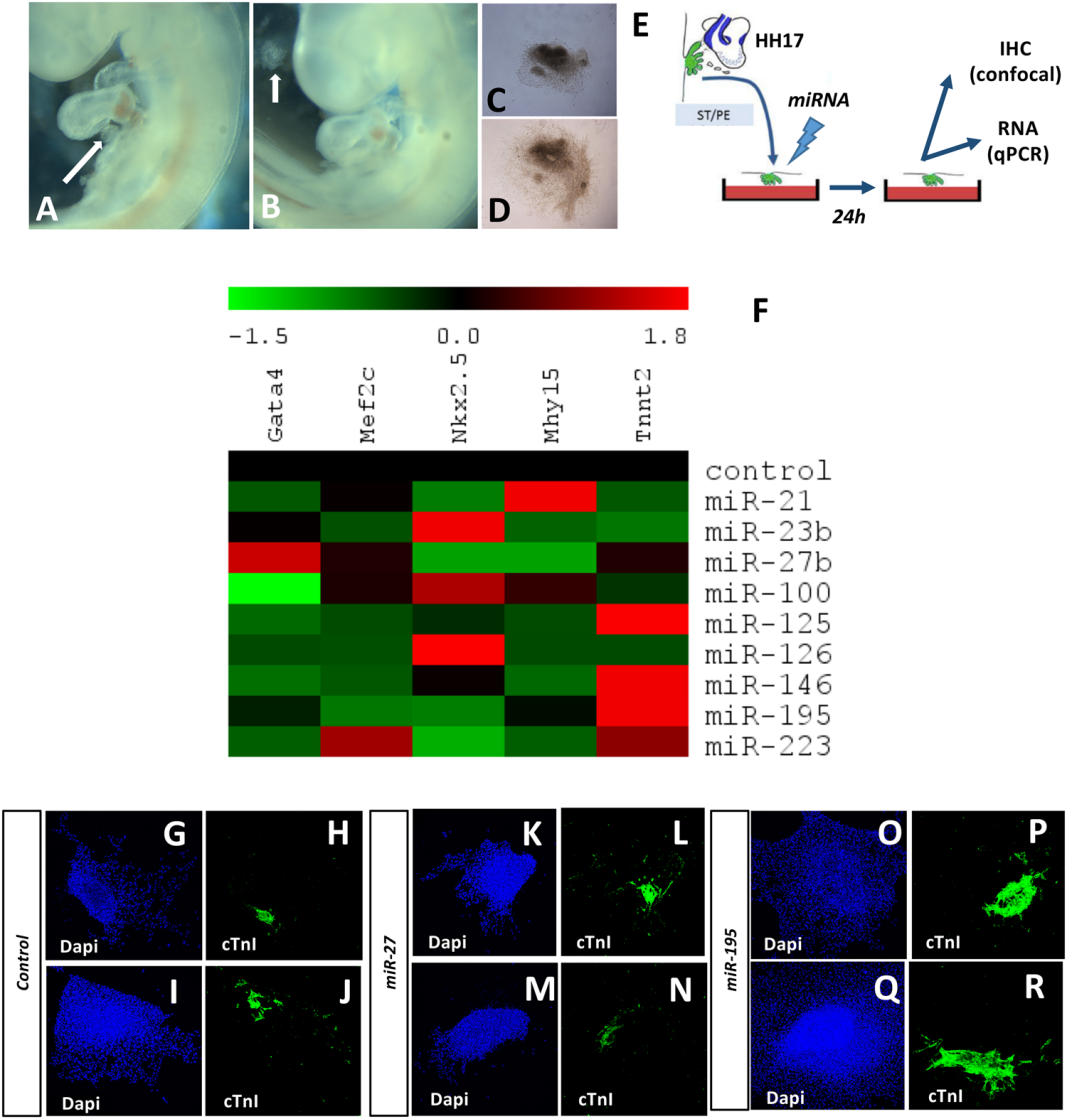
Modulation of cardiomyogenic potential of PE/ST explants by microRNA mimics administration Panels A–D. Representative image of chicken HH17 embryo before (panel A) and after (panel B) PE excision. Arrows demarcates the PE. Panel C illustrates PE culturing just right after dissection (panel C) and 24 h after culturing (panel D). Panel E represents an schematic overview of the experimental design. Panel F shows qPCR results of cardiomyogenic (*Nkx2.5, Mef2c, Gata4, Mhy15, Tnnt2*) markers expression after microRNA mimic administration in HH17 PE/ST explants. Observe that miR-23 and miR-27 over-expression leads to down-regulation of all cardiomyogenic markers, miR-100 does not modify most of them and miR-223, miR-195, miR-125 and miR-146 increased terminally differentiation markers such as cardiac troponin T (*Tnnt2*). Confocal image analyses of cTnI expression in controls (panels G-J), miR-27 (panels K-N) and miR-195 (panels O-R) treated HH17 PE/ST explants. Observe that miR-195 administration selectively increases the overall cTnI immunohistochemical signal (panel P and R). HH17 PE were dissected from >30 embryos, treated with the corresponding microRNA mimics and subsequently pooled to perform RNA isolation. On each case, three-to-five distinct biological replicates were subsequently tested by qPCR.

microRNAs play modulatory roles in multiple aspects of embryonic development and therefore also during cardiac formation. We have previously reported a pivotal role for miR-23b and miR-199 during epithelial-to-mesenchymal transition in cardiac valvulogenesis^28^. We herein tested whether microRNA administration can influence cell lineage determination of the PE/ST, focusing particularly on cardiomyogenic differentiation. Chicken HH17 PE/ST explants were isolated **(**Fig. 2A–E**)** and cultured in collagen gels as previously described^28^. Treatment of the PE/ST explants was carried with nine distinct microRNAs (*miR-21, miR-23b, miR-27b, miR-100, miR-125, miR-126, miR-146, miR-195* and *miR-223*), representing microRNAs that display distinct expression profiles during embryonic epicardial development (i.e. within the four distinct categories previously described), for 48 hrs and tissues were either processed for qPCR or immunohistochemistry and confocal image analyses, respectively. In all cases, at least 30 PE/ST explants were treated for each experimental conditions that were subsequently pooled. qPCR analyses was performed for markers of early (*Mef2c, Nkx2.5 and Gata4*) and terminal cardiogenic differentiation (*Mhy15* and *Tnnt2*), epithelial to mesenchymal transition (*Snail, Slug, Cdh1, Cdh2, Cdh5*), and fibrogenesis (*Col1a1*). Analyses were always performed in 3–5 distinct biological replicates for each microRNA treatment. Overexpression of microRNA mimics were validated by qPCR as illustrated in Supplementary Fig. 1. Expression of early cardiomyogenic differentiation markers such as *Mef2c, Nkx2.5* and/or *Gata4* were selectively down-regulated (or not altered) by *miR-21, miR-23b, miR-27b, miR-100, miR-195, miR-125* while *miR-126* and *miR-146*, respectively, enhanced expression of *Nkx2.5* and *miR-223* administration increased expression of all three early cardiomyogenic markers. Cardiogenic terminal differentiation markers were significantly up-regulated in PE/ST treated with *miR-223, miR-195, miR-125 and miR-146*, respectively while they were selectively inhibited by *miR-23b, miR-27b* and *miR-126* but not significantly altered by *miR-100* and *miR-21* administration (Fig. 2F). These data were further corroborated by immunohistochemical detection of cardiac troponin I as illustrated in Fig. 2G–R. Curiously, in most cases that terminal differentiation is enhanced, a selective down-regulation of most of the early cardiogenic markers is observed (*miR-195, miR-125* and *miR-146*), except for *miR-223* that were equally enhanced.

PE/ST cells migrate into the nude myocardium and activate an epithelial-to-mesenchymal transition that is pivotal for it subsequent migration into the embryonic myocardium. However, it is unclear if EMT is required for PE/ST differentiation. Thus, we tested if EMT also display significant differences after microRNA administration, particularly those promoting cardiomyogenesis. *miR-195* and *miR-23* treatment decreased EMT markers such as *Snail* and/or *Slug*, whereas *miR-27b, miR-21, miR-100, miR-146, miR-125, miR-126* and *miR-223* increased those EMT transcriptional activators (Supplementary Fig. 2). Our data support the notion that EMT and cardiomyogenic differentiation are not mutually exclusive biological processes, i.e. both can concur simultaneously. Curiously, expression of cell-cell junctional proteins, i.e. *Cdh1, Chd2* and *Chd5*, was not always concomitantly up-regulated or down-regulated as the EMT transcriptional activators (Supplementary Fig. 2), suggesting that different molecular mechanisms drive activation and cell-cell uncoupling, in line with previous reports^28^ and/or that transcriptional overriding after microRNA administration is operative as suggested by Hill *et al*.^37^.

Fibrogenic deposition is a major hurdle for proper cardiomyocyte functional electrical coupling. We therefore tested if microRNAs promoting cardiomyogenesis would be concomitantly increasing fibrogenic differentiation. Our data demonstrate that fibrogenic differentiation, as assessed by *Col1a1* expression, was significantly increased after *miR-23b*, *miR-27b*, *miR-195 and miR-223*, administration whereas *miR-100* and *miR-125* treatment decreased *Col1a1* expression (Supplementary Fig. 2). No significant changes were observed for *miR-21, miR-146* and *miR-126* administration, respectively (Supplementary Fig. 2). Overall these data demonstrate that microRNA treatment can distinctly modulate cell differentiation behavior including cardiomyocyte, epithelial to mesenchymal transition and fibroblast differentiation.

In sum, these data demonstrate that single microRNA administration can exert different cell differentiation modulatory roles; e.g. miR-23 inhibits cardiogenesis and epithelial-mesenchymal transition while enhances fibrogenesis while miR-195 enhances terminal cardiomyocyte differentiation and fibrosis while inhibits epithelial-to-mesenchymal transition.

### Modulatory effects of miR-195 and miR-233 is partially promoted in the embryonic epicardium

**Figure 3:**
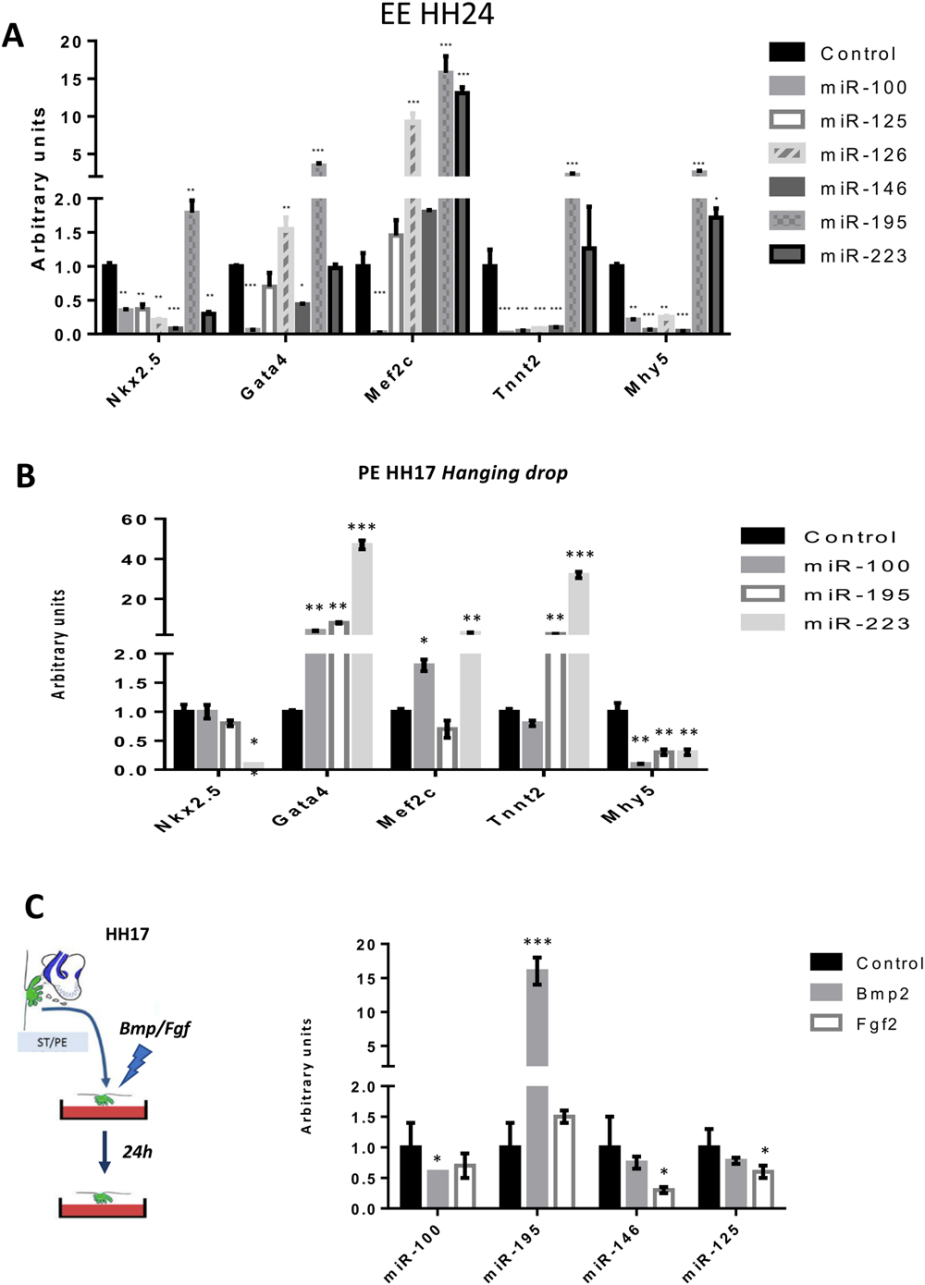
Modulation of cardiomyogenic potential of HH24 embryonic epicardium and HH17 PE/ST in hanging drops by microRNA mimics administration. Modulation of microRNAs by Bmp and Fgf signaling. Panel A represents qPCR analyses of HH24 embryonic epicardium treated with microRNA mimics. Observe that miR-195 and miR-223 selectively enhance expression of terminally differentiation markers such as *Tnnt2* and *Mhy15*. Panel B represents qPCR analyses of HH17 PE/ST explants cultured in handing drops and treated with microRNA mimics. Observe that miR-195 and miR-223 selectively enhance expression of terminally differentiation marker *Tnnt2*, while administration of miR-100 does not changes it expression. Panel C qPCR analyses of HH17 PE/ST explants cultured in handing drops and treated with Bmp2 and Fgf2, respectively. Observe that Bmp2 selectively enhances expression of miR-195 while no significant differences are observed after Fgf2 administration. HH17 PE were dissected from >30 embryos, treated with the corresponding microRNA mimics and/or Bmp/Fgf treatment, respectively, and subsequently pooled to perform RNA isolation. On each case, three-to-five distinct biological replicates were subsequently tested by qPCR.

In order to dissect if the modulatory roles exerted by distinct microRNAs in the HH17 PE/ST explants is also applicable to the embryonic epicardium, HH24 epicardial explants were analyzed after microRNA over-expression of a selected number of microRNAs, i.e. those reporting enhanced cardiomyogenesis *miR-125, miR-126, miR-146, miR-195* and *miR-223*, and *miR-100* as a negative control. Regulation of early myogenic markers is partially discordant as compared to PE/ST explants. For example, *miR-100*, *miR-125* and *miR-126* administration displayed decreased *Nkx2.5* expression in EE HH24 cells while no changes or increased is observed in HH17 PE/ST explants **(**Fig. 3A**)**. On the other hand, concordant modulation is observed for *Gata4* after *miR-100, miR-125* and *miR-146*, as well as for *Mef2c* after *miR-125, miR-146* and *miR-223* **(**Fig. 3A**)**. Importantly, up-regulation of terminally differentiation markers such as *Tnnt2* and *Mhy5* as also concordantly observed after *miR-195* and *miR-223*, demonstrating that cardiomyogenesis is similarly enhanced in EE HH24 and PE/ST explants.

Epithelial-to mesenchymal transition also display concordant expression for *Slug* following microRNA administration in HH24 embryonic epicardial cells, in particular for *miR-100, miR-125, miR-126* and *miR-146* while opposite regulatory patterns are observed after *miR-195* and *miR-223* administration, respectively **(**Supplementary Fig. 2**)**. Similar discordant patterns are observed for *Cdh5* expression except for *miR-223* **(**Supplementary Fig. 2**)**. Curiously, no significant difference in cell migratory behavior was observed in time-lapse experiments (data not shown).

Expression of the fibrogenic marker *Col1a1* display similar concordant patterns in EE HH24 and PE/ST explants after administration of *miR-100* and *miR-125*, whereas discordant patterns were observed for all the other microRNAs tested **(**Supplementary Fig. 2**)**. Overall these data demonstrate that *miR-195* and *miR-223* maintain their potentially to enhance cardiomyogenesis in the embryonic epicardium and while the loose their ability to promote epithelial-to mesenchymal transition and enhance fibrogenesis in EE HH24 as compared to PE/ST explants. Thus, these data suggest a plausible therapeutic usage of *miR-195* and *miR-223* to enhance cardiomyogenesis without compromising putative adverse events such as EMT and fibrosis promotion.

In addition, we have also tested if these modulatory effects were similarly occurring in the PE HH17 cultured in hanging drop, to dissect if cell-matrix interactions are required or not for microRNA-mediated cardiac differentiation. For this purpose we assayed only those microRNAs displaying enhanced cardiomyogenesis in PE/ST explants and HH24 EE cultures, i.e. *miR-195* and *miR-223*, and *miR-100* as a negative control. Briefly, PE HH17 were dissected, set into hanging drops and concomitantly transfected with distinct microRNA mimics. After 24 h, RNA was isolated and cell lineage markers were assessed by qPCR. Our data demonstrate that administration of *miR-100* does not enhance the expression of terminally differentiation cardiomyogenic marker cardiac troponin T while *miR-195* and *miR-223* significantly increased it **(**Fig. 3B), in line with previous data in PE HH17 cultured in collagen matrices (Fig. 2F) and HH24 EE cell cultures (Fig. 3A). Surprisingly, no enhancement was observed for Mhy5, probably due do time-specific differences in the onset of expression of these cardiomyogenic markers. Thus, our data revealed that cell-matrix interactions are not required for microRNA-mediated cardiomyogenesis.

### Bmp and Fgf can distinctly modulate microRNA expression in the developing proepicardium

Several growth factors members of the BMP and FGF families have been reported to distinctly modulate cell lineage specification in cardiogenic mesoderm into proepicardial and myocardial cells, respectively^14^. In particular, Bmp2 promotes differentiation of the septum transversum mesoderm into myocardial cells whereas Fgf2 enhances proepicardial lineage commitment, a process that is intricately regulated by a complex feed-back loop involving several other Bmp and Fgf family members^14^. We experimentally tested whether Bmp and Fgf signaling in the developing PE/ST influence the expression of distinct microRNAs with potential to modulate PE/ST cell differentiation as reported above. HH17 PE/ST tissues were dissected and cultured in hanging drops. Administration of Bmp2 significantly increased expression of *miR-195* while blocked expression of *miR-100* and no significant differences were observed for *miR-146* and *miR-125*. On the other hand, Fgf2 administration selectively blocked *miR-125, miR-100, miR-125, miR-146* but enhanced *miR-195* expression (Fig. 3C). These data illustrate that distinct administration of Bmp and Fgf signaling influence miRNA expression in the developing PE/ST tissues.

### Search for common miRNA-mRNA pathways modulating cardiogenic lineage commitment

**Figure 4:**
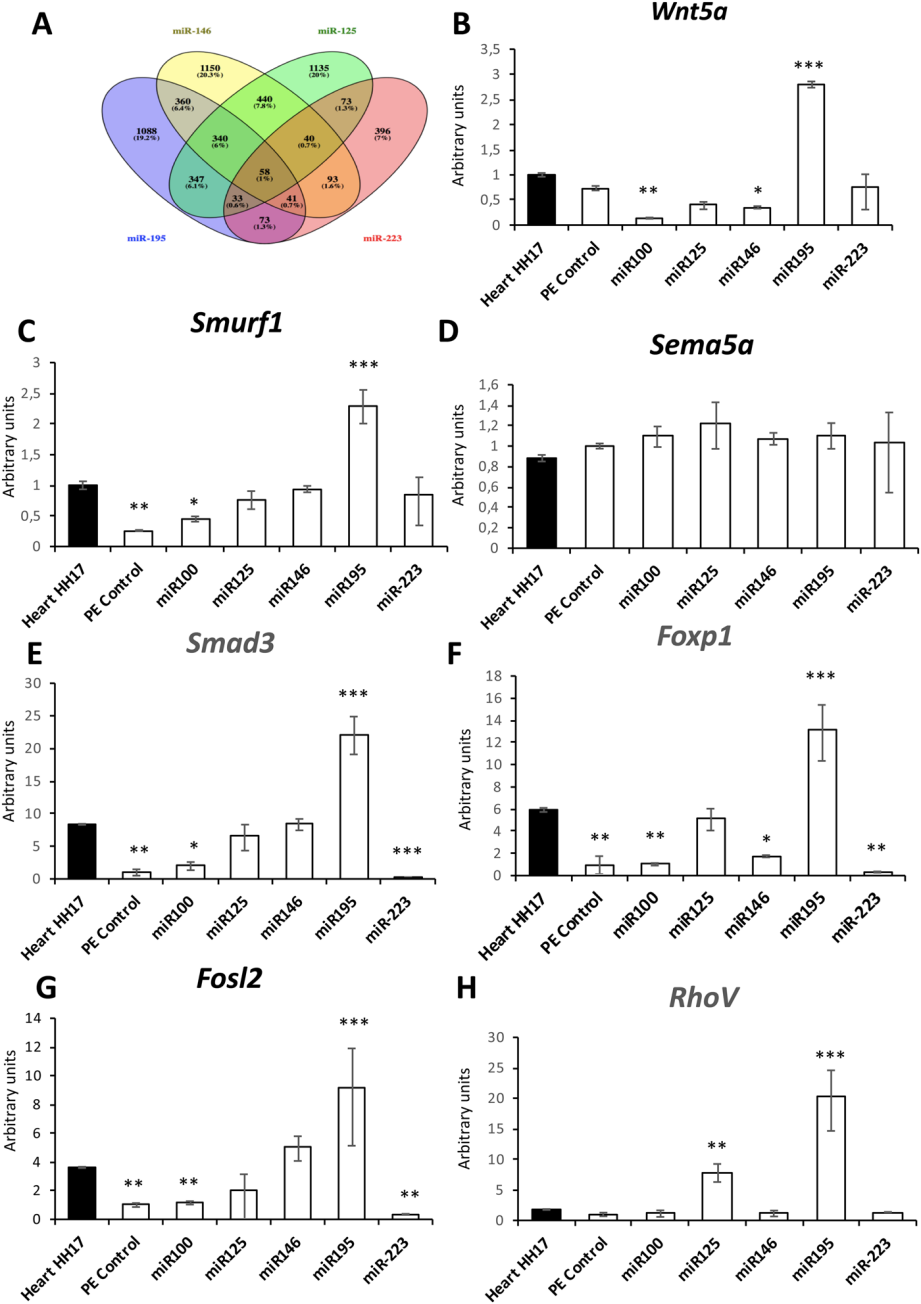
Potential targets driving PE/ST explants cardiomyogenesis. Panel A. Venny diagram of shared potential targets of miR-146, miR126, miR-195 and miR-223. Observe that only 58 potential target (representing 1%) were shared between these four microRNAs. Panels B-H. qPCR analyses of the expression of *Wnt5a* (panel B), *Smurf1* (panel C), *Sema5a* (panel D), *Smad3* (panel E), *Foxp1* (panel F), *Fols2* (panel G) and *RhoV* (panel H) in HH17 hearts, HH17 control PE/ST explants and PE/ST explants treated with miR-100, miR-125, miR-146, miR-195 and miR-223. Observe that over-expression of miR-195 increases expression of *Wnt5a, Smurf1, Smad3, Foxp1, Fols2* and *RhoV*, while no significant differences were observed for *Sema5a*. On the contrary, miR-100 over-expression lead to significant downregulation of *Wnt5a, Smurf1*, up-regulation of *Smad3* and no significant differences of *Sema5a, Foxp1, Fosl2* and *RhoV*. HH17 PE were dissected from >30 embryos, treated with the correspoding microRNA mimics and subsequently pooled to performed RNA isolation. On each case, three distinct biological replicates were subsequently tested by qPCR.

microRNAs can modulate multiple mRNA transcripts, ranging from hundreds to thousands targets^38^. Distinct *in silico* algorisms can predict micro-mRNA interaction based on sequence complementary, biophysical interaction models and evolutionary conservation (i.e. TargetScan; http://www.targetscan.org/vert_72/ and MirWalk; http://mirwalk.umm.uni-heidelberg.de). We have demonstrated that over-expression of *miR-195, miR-125, miR-146* and *miR-223* respectively, in HH17 PE explants can enhance cardiomyocyte terminal differentiation. We therefore thought that they might share common targets governing these phenotypic changes. We selected all putative mRNA targets of *miR-195, miR-125, miR-146* and *miR-223* using MirWalk software and we identified all shared targets between these microRNAs. A total of 58 (1% all putative targets) mRNAs were identified for all four microRNAs, while 454 (8% all putative targets) were shared in three out of four microRNAs (Fig. 4A). We subsequently scrutinized all genes (512 target genes; 9% all putative targets) with previous cited reports playing a role in myogenesis, and selected short list of seven transcripts (*Wnt5a, Smurf1, Sema5a, Smad3, Foxp1, Fosl2, RhoV*) for further testing their implication in PE/ST-derived cardiomyogenesis. We then tested if these genes were modulated by *miR-195, miR-125, miR-146* or *miR-223* over-expression, respectively, in HH17 PE explants. In addition, *miR-100* over-expression was also assayed as a negative control of cardiomyogenic inhibition and HH17 embryonic heart expression was also included to compare the relative expression of these genes in proepicardial and myocardial cells.

Comparative analyses of shared targets in the HH17 PE and embryonic heart demonstrate that *Smurf1, Smad3, Foxp1, Fosl2* are enriched in the embryonic heart as compared to the PE, whereas *Wnt5a Sema5a* and *RhoV* display no significant differences (Fig. 4B–H). Over-expression of miR-100 selectively increased *Smad3*, decreased *Wnt5a* and *Smurf1* while no significant differences were observed for *Sema5a, Foxp1, Fosl2* and *RhoV* (Fig. 4B–H). Administration of *miR-125* resulted in down-regulation of Wnt5a, up-regulation of *Smad3, Foxp1* and *RhoV*, while no significant differences were observed for *Smurf1*, *Sema5a* and *Fols2*. Similarly, *miR-146* over-expression resulted in down-regulation of *Wnt5a*, up-regulation of *Fosl2*, whereas all the other putative targets display no changes (Fig. 4B–H). Administration of *miR-195* to HH17 PE explants resulted in up-regulation of *Wnt5a, Smurf1*, *Smad3*, *Foxp1, Fosl2* and *RhoV*, but no significant changes were detected for *Sema5a* (Fig. 4B–H). Overall these data demonstrated that most of the shared targets are enriched in the HH17 PE. In addition, *miR-125, miR-146* and *miR-195* selectively modulate expression of these genes as predicted, except for *Sema5a*. Furthermore our data illustrate that *miR-195* exerts up-regulation of multiple genes involved in early cardiomyogenesis, in line with our results demonstrating that *miR-195* over-expression in HH17 PE explants enhances cardiomyogenesis, whereas *miR-100* blocks several of them, in line with HH17 PE explants over-expression assays. Among those shared targets, up-regulation of *Fosl2* and *Smad3* is exerted by both *miR-146* and *miR-195*, whereas up-regulation of *RhoV* and *Foxp1* is exerted by *miR-125* and *miR-195*, and *Wnt5a* and *Smurf1* by *miR-195* posing those genes as good candidates to explain the phenotypic consequences of driving cardiomyogenic differentiation upon microRNA over-expression.

### Smad3 and Smurf1, but not Fols2, are essential for miR-195 driven cardiomyogenesis in PE/ST explants

**Figure 5:**
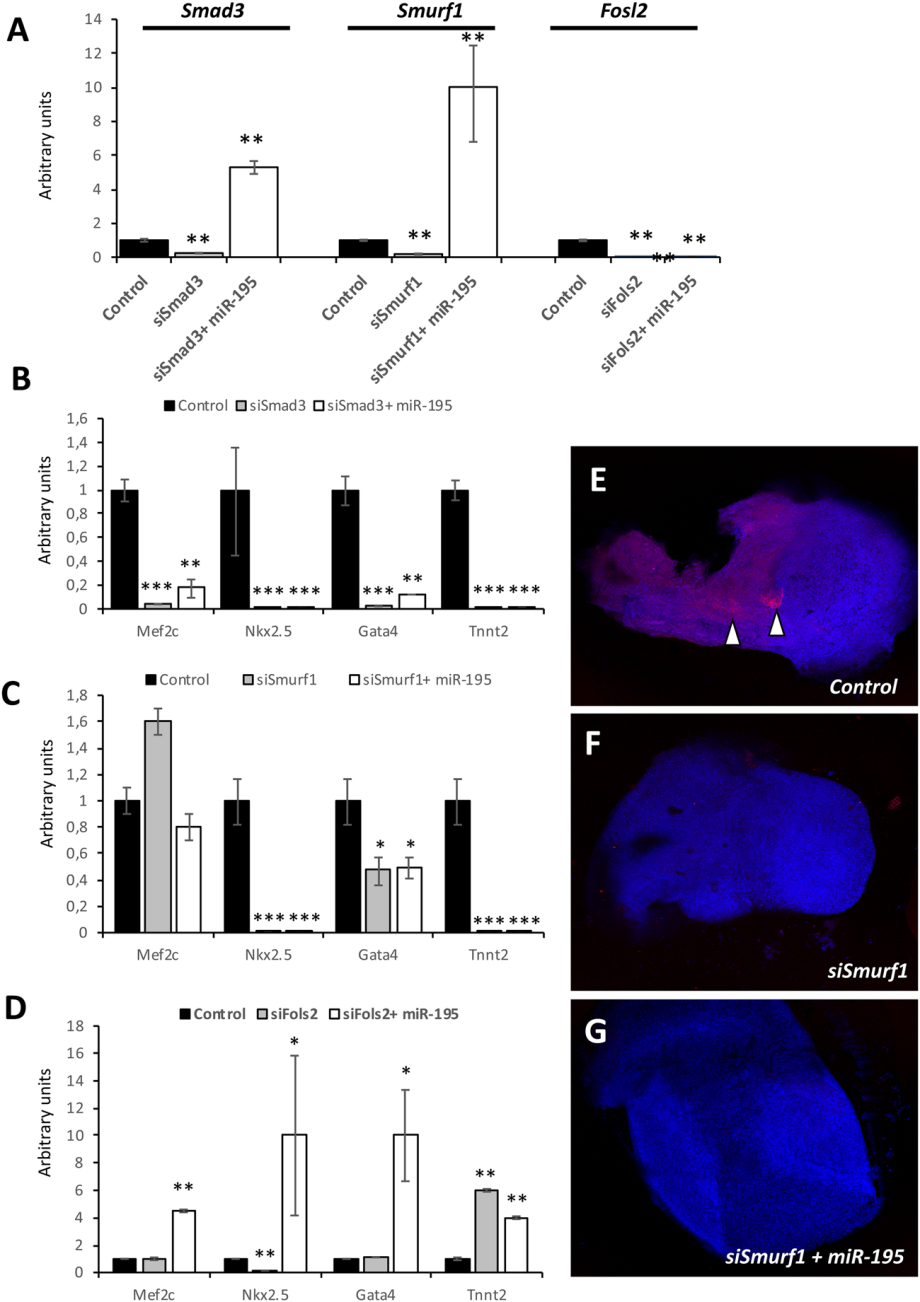
Smad3 and Smurf1, but not Fols2, are essential for miR-195 driven cardiomyogenesis in PE/ST explants. Panel A. qPCR analyses of *Smad3, Smurf1* and *Fols2* in control, siRNA treated and siRNA plus miR-195 overexpression. Observe that siRNAs against *Smad3, Smurf1* and *Fols2* significantly decrease *Smad3, Smurf1* and *Fols2*, respectively. Observe also that miR-195 administration can rescue *Smad3* and *Smurf1* up-regulation but not *Fols2*. Panel B. qPCR analyses of cardiomyogenic markers in control, siSmad3 treated and siSmad3 plus miR-195 overexpression. Observe that expression of *Mef2c, Nkx2.5, Gata4* and *Tnnt2* is severely impaired in both siSmad3 treated and siSmad3 plus miR-195 overexpression conditions. Panel C. qPCR analyses of cardiomyogenic markers in control, siSmurf1 treated and siSmurf1 plus miR-195 overexpression. Observe that expression of *Nkx2.5, Gata4* and *Tnnt2* is severely impaired in both siSmurf1 treated and siSmurf1 plus miR-195 overexpression conditions. Panel D. qPCR analyses of cardiomyogenic markers in control, siFols2 treated and siFols2 plus miR-195 overexpression. Observe that expression of *Mef2c, Gata4* is not altered, *Nkx2.5* is diminished but *Tnnt2* is significantly increased in siSmurf1 conditions. Furthermore, *Mef2c, Nkx2.5, Gata4* and *Tnnt2* expression is significantly increased in siSmurf1 plus miR-195 overexpression conditions. HH17 PE were dissected from >30 embryos, treated with the corresponding microRNA mimics, siRNA or the combination of both (microRNA mimic and siRNA) and subsequently pooled to performed RNA isolation. On each case, three distinct biological replicates were subsequently tested by qPCR.

In order to test the functional role of these genes in miR-195 promoted cardiomyogenesis, loss-of-function experiments were performed in presence or absence of miR-195. siRNAs were successfully designed and validated against *Fols2, Smad3* and *Smurf1* while failed for *Wnt5a* and *Foxp1* silencing either on the design itself or validation (data not shown). As illustrated in Fig. 5A, successful inhibition was obtained for *Smad3, Smurf1* and *Fols2*, respectively. Interestingly, co-administration of miR-195 mimics and the corresponding siRNA, rescued *Smad3* and *Smurf1* but not *Fols2* expression. Subsequently we tested if siRNA silencing leads to impair cardiomyogenesis in PE/ST explants and if silencing was rescued by *miR-195* administration by measuring early (*Mef2c, Gata4* and *Nkx2.5*) and terminally (*Tnnt2*) differentiation cardiomyocyte markers by qPCR. Our data demonstrate that *Smad3* silencing significantly blocked the expression of *Mef2c, Gata4, Nkx2.5* and *Tnnt2*, while *miR-195* administration in this setting of Smad3 silencing only partially rescued expression of *Nkx2.5* and *Gata4* but without reaching control levels (Fig. 5B). Similarly, *Smurf1* siRNA significantly down-regulates *Nkx2.5, Gata4* and *Tnnt2*, but surprisingly up-regulates *Mef2c*. *miR-195* administration on *Smurf1* siRNA treated explants partially rescued *Mef2c* to basal control levels but it was unable to recover *Nkx2.5, Gata4* and *Tnnt2* (Fig. 5C). Immunohistochemical analyses against cardiac troponin T corroborated these findings (Fig. 5E–G). Finally, *Fols2* siRNA display no significant differences on *Mef2c* and *Gata4* expression while *Nkx2.5* was decreased and *Tnnt2* was increased. Moreover, *miR-195* administration in *Fols2* siRNA treated PE/ST explants significantly up-regulated all cardiomyogenic markers (Fig. 5D). These data demonstrate that *Smad3* and *Smurf1* expression is critical for enhanced cardiomyogenesis in PE/ST explants while *Fols2* seems to be dispensable.

### Bmp4_53170 and Fgf8_57126 are modulated by miR-195 in PE/ST explants

**Figure 6:**
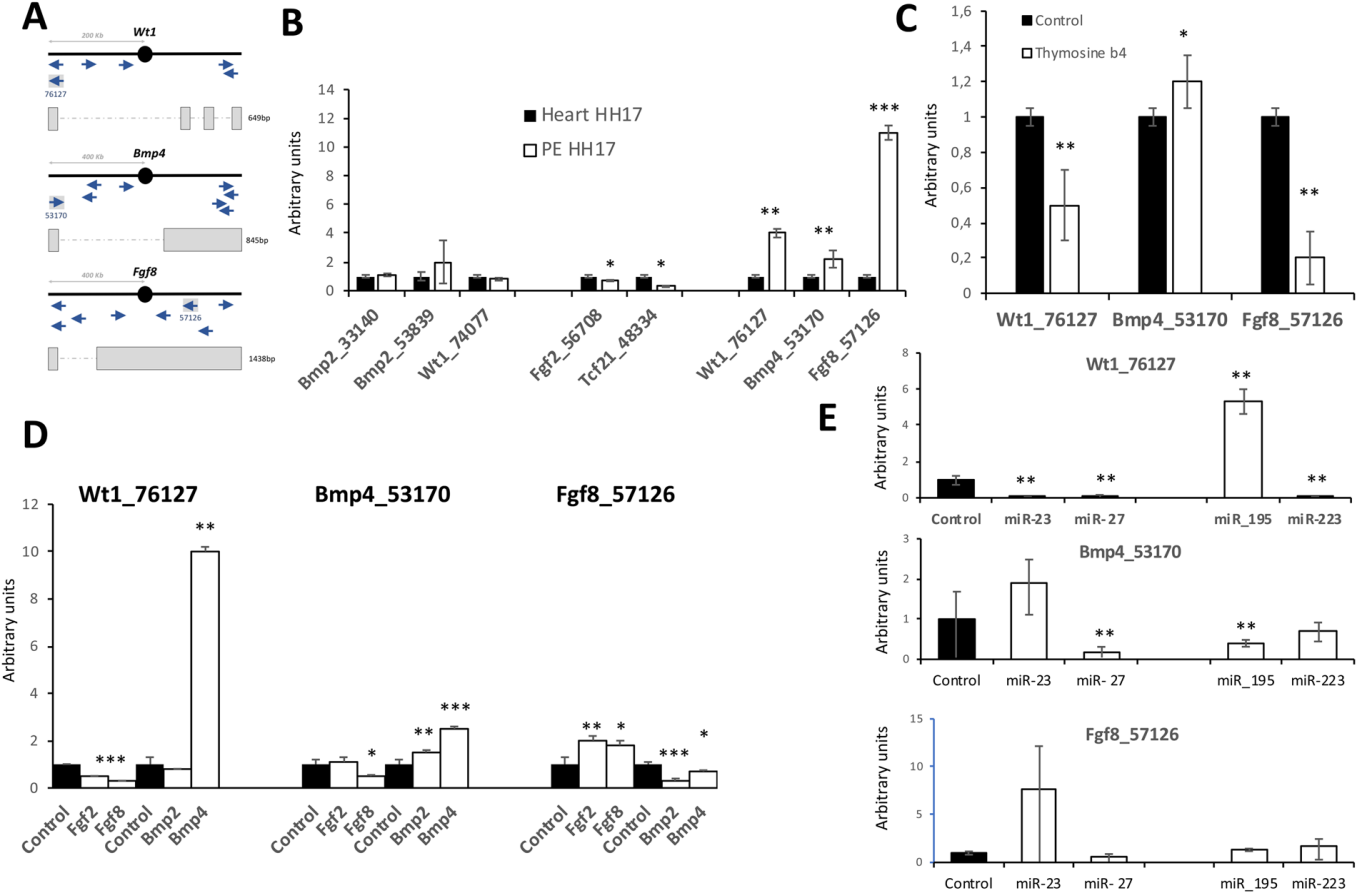
LcnRNA expression and modulation in PE/ST explants Panel A. Schematic representation of the genomic location of annotated lncRNAs within Wt1, Bmp4 and Fgf8 loci in chicken. Panel B. qPCR analyses of the expression of lncRNAs in HH17 embryonic heart and PE/ST, respectively. Observe that Wt1_76127, Bmp4_53170 and Fgf8_57126 display significant increased expression in the PE/ST as compared to the embryonic heart at the same developmental stage (HH17). Panel C. qPCR analyses of Wt1_76127, Bmp4_53170 and Fgf8_57126 in thymosine β4 treated PE/ST explants. Observe that thymosine β4 treatment increases Bmp4_53170 while decreased Wt1_76127 and Fgf8_57126 expression. Panel D. qPCR analyses of Wt1_76127, Bmp4_53170 and Fgf8_57126 in Bmp2, Bmp4, Fgf2 and Fgf8 treated PE/ST explants, respectively. Observe that Bmp signaling decreased while Fgf signaling increased the expression of Wt1_76127, Bmp4_53170, while Fgf8_57126 displays the opposite regulatory modulation by Bmp and Fgf signaling. Observe that miR-195 increases Wt1_76127 while decreases Bmp4_53170 expression. On the other hand miR-23 and miR-27 decreases Wt1_76127 while does not modify the expression of Bmp4_53170. Panel E. qPCR analyses of Wt1_76127, Bmp4_53170 and Fgf8_57126 in miR-23, miR-27, miR-195 and miR-223 treated PE/ST explants, respectively. HH17 PE and HH17 embryonic hearts were collected from >30 embryos and pooled to performed RNA isolation. In all cases, three distinct biological replicates of pooled HH17 PE/ST and HH17 heart samples were subsequently tested by qPCR (panel A). HH17 PE/ST were dissected from >30 embryos, treated with thymosine beta4 (panel C), the correspoding Bmp/Fgf growth factor (panel D), and/or microRNA mimics treatment (panel E), respectively and subsequently pooled to performed RNA isolation. On each case, three distinct biological replicates were subsequently tested by qPCR.

Long non coding RNAs are emerging as novel molecules playing essential roles in gene expression regulation in multiple biological contexts^39–41^. Several studies have provided evidence that lncRNAs can play regulatory roles affecting neighboring genes^42–44^. We have identified nine distinct annotated lncRNAs neighboring key regulatory factors (*Bmp2, Bmp4, Wt1, Fgf2, Fgf8, Tcf21*) involved in PE development in the chicken genome (Fig. 6A) and we have assessed their expression in PE as compared to age-matched developing heart. Three distinct patterns were observed, those displaying no significant differences (*Bmp2_33140, Bmp2_53839, Wt1_74077*), those with decreased expression in PE (*Fgf2_56708, Tcf21_48334*) and those with increased expression in PE (*Wt1_76127, Bmp4_53170* and *Fgf8_57126*) as compared to HH17 embryonic heart (Fig. 6B). We subsequently assessed if those lncRNAs with enhanced expression in PE were modulated by cardiomyogenic enhancing signals provided by Bmp administration or PE signals provided by Fgf administration^14^. We observed that *Wt1_76127* was significantly down-regulated by Fgf8 while up-regulated by Bmp4 administration. Similarly, *Bmp4_53170* was down-regulated by Fgf8 while up-regulated by both Bmp2 and Bmp4. On the other hand, *Fgf8_57126* was up-regulated by Fgf2 and Fgf8 while significantly down-regulated by Bmp2 and Bmp4 (Fig. 6D). Overall these data demonstrate that these lncRNAs are complementary modulate by Fgf and Bmp signaling suggesting a plausible role in PE/ST derived cardiomyogenesis. In addition, we also tested if thymosin β4, a epicardial to myocardial priming agent^15^ could modulate the expression these lncRNAs. Interestingly, thymosin β4 enhanced *Bmp4_53170* while inhibited *Wt1_76127* and *Fgf8_57126* expression in PE/ST explants (Fig. 6C), further supporting a plausible role in PE/ST derived cardiomyogenesis, yet additional experiments are required to fully understand their functional role in this context.

Given the plausible role of these lncRNAs in PE/ST derived cardiomyogenesis, we tested if those microRNAs enhancing (*miR-195* and *miR-223*) or blocking (*miR-23* and *miR-27*) cardiomyogenesis are capable of modulating their expression. PE/ST explants treated with *miR-23* and *miR-27* significantly inhibited expression of *Wt1_76127*, but did not modify expression of *Bmp4_53170* and *Fgf_57126*. On the other hand, miR-195 significantly enhanced *Wt1_76127* while inhibited *Bmp4_53170*, but no differences were observed for *Fgf8_57126*. miR-223 administration significantly blocked *Wt1_76127* while *Bmp4_53170* and *Fgf8_57126* display no significant differences (Fig. 6E). These data demonstrate that microRNAs can regulate these lncRNAs and furthermore, microRNAs promoting vs inhibiting cardiomyogenesis display complementary regulatory roles, particularly on *Wt1_76127* and *Bmp4_53170*, further reinforcing their plausible role in PE/ST derived cardiomyogenesis.

## Discussion

Differential expression of microRNAs have been widely reported in distinct biological settings including homeostatic and pathological contexts^45–47^. Within the cardiovascular system, several studies have provided evidences of the differential expression of microRNAs during cardiogenesis^48,49^. However, to date, microRNA profiling of the proepicardium and/or epicardium is still missing. We provide herein evidence that multiple microRNAs display differential expression during the process of PE and epicardium formation. A large subset of microRNAs display increasing expression, supporting a plausible role blocking or inhibiting the expression of mRNA target genes during PE to embryonic epicardial transition. On the other hand, a small subset display decreased expression supporting a role in releasing repression of inductive signals while a similar subset display transition peak expression in HH24 as compared to HH32 embryonic epicardium, suggesting a plausible modulatory role in this transition, probably affecting thus epicardial to mesenchymal transition onset^50–52^. Thus, these data provide an entry site to start dissecting the functional roles of microRNAs during epicardial development.

Seminal evidences on the functional role of microRNAs in epicardial development was provided by Singh *et al*.^25^ by selective deletion of *Dicer*, a ribonuclease involved in microRNA maturation, in the embryonic epicardium. However, understanding of the functional role of discrete microRNAs in the epicardium have only been provided for miR-31 and miR-21, both of them directing fibrogenic EMT by distinctly modulating *Islet1*^51^ and *Pcd4*/*Spry1*^53^ expression, respectively. Importantly, to the best of our knowledge this is first evidence reporting cardiomyocyte cell fate modulation of the PE/ST. A significant enhancement of cardiomyocyte terminal differentiation was provided by administration of *miR-223* and *miR-195* mimics, a weaker activation was provided by *miR-125* and *miR-146* while only activation of early cardiogenic markers but not terminal differentiation was obtained for *miR-126*. On the other hand, *miR-23* and *miR-27* selectively inhibited cardiomyogenesis while *miR-100* and *miR-21* essentially displayed not significant enhancement. These data therefore evidence the differential microRNA modulation of PE/ST cardiomyogenesis.

Previous studies reported the involvement of *miR-23* and *miR-27* in both cardiac development and pathology^48,54–58^, while *miR-100* has only been reported as a protective agent of cardiomyocyte apoptosis^59^. On the other hand, *miR-223* and *miR-195* have been reported in distinct cardiac pathologies^60–67^ but no evidences on their functional role during cardiac development have been described so far. Furthermore, scarce evidences on the role of *miR-125*^68^ and/or *miR-14*^69^ in cardiac development and pathology have been reported. On the other hand, *miR-126* represents a vascular specific microRNA and *miR-126* deficient zebrafish are embryonic lethal^70^. Furthermore, additional functional roles for miR-126 in the vasculature have been extensively reported^71–74^. Importantly a functional role in cardiomyocytes, particularly in apoptosis, is recently emerging^75–77^. Our data demonstrate that a more enhanced cardiomyogenic differentiation is exerted by miR-195 and miR-223 as compared to miR-125, miR-146 and miR-126, while miR-23 and miR-27 blocked such cardiomyogenic differentiation. Furthermore, our findings open up the possibility of exploring these microRNAs as therapeutic tools to enhance cardiomyogenesis.

Regulation of cardiac transcription factors such as *Mef2c*, *Gata4* and *Nkx2.5* by microRNAs have been reported in different biological contexts^78–80^. In striated muscle, *miR-27* and *miR-125* distinctly regulate *Mef2c* in cardiac and skeletal muscle cells^31,48^. Curiously, miR-223 downregulation leads to *Mef2c* upregulation in leukemia^81^ while no evidences have been reported for miR-195 and/or miR-146 modulating the expression of these early cardiomyogenic differentiation markers in striated muscle. In this study we demonstrate for the first time the regulatory role of these microRNAs modulating expression of early and terminally differentiation cardiomyogenic markers in both PE/ST and epicardial cell cultures, enhancing thus their potential therapeutic usage.

An integral developmental process linked to PE and epicardium morphogenesis is driven by an epithelial to mesenchymal transition that provides mechanistic clues to these cells favouring their integration into the embryonic myocardium and subsequently differentiation into distinct cell types, such as fibroblasts smooth muscle cells and endothelial cells^52^. In this study we further investigated how administration of these microRNAs influence EMT and fibrogenic differentiation. Our data demonstrate that *miR-195* and *miR-23* can selectively down-regulate expression of EMT inducers such as *Snail* and *Slug* and up-regulate *Cdh1* expression without modulating *Cdh2* and *Chd5*, in line with previous reports in other biological contexts^72,82^. On the other hand, all the other microRNAs tested (i.e. *miR-21, miR-27, miR-100, miR-125, miR-126, miR-223* and *miR-146*) resulted in *Snail* and/or *Slug* up-regulation while effects on *Cdh* expression is not always concomitant. Several of these microRNAs have been reported to promote EMT^83,84^ while other can either promote or inhibit it in different biological contexts^85–94^, in line with our findings. Importantly, we provide evidence for the first time on the involvement of *miR-125* and *miR-146* in EMT regulation. Our data suggest that up-regulation of EMT inducers and subsequent cytoskeletal remodeling represent uncoupled events in this setting, in line with previous reports during AV EMT modulation by microRNAs^28^, alternatively that additional time is required to see such transcriptional changes in *Cdh* expression or that transcriptional overriding effects by microRNAs over-expression is occurring^37^. Thus, additional experiments are required to fully elucidate this apparently discordant findings. Furthermore, our data demonstrate that EMT is not required to PE/ST cardiomyogenic differentiation, since administration of *miR-223* can simultaneously induce both processes. i.e. EMT and cardiomyogenesis.

Importantly, we demonstrate herein that a single microRNA can exert different regulatory aspects in both PE/ST explants and HH24 EE cell cultures. *miR-23* can block cardiomyogenic differentiation and EMT while promotes fibrogenic differentiation, *miR-195* enhances cardiomyogenesis while blocking EMT but promoting fibrogenic differentiation while, *miR-223* can promote all three developmental processes, providing thus a therapeutic potential for cardiomyogenic regeneration.

To understand the molecular mechanisms that drive promotion of cardiomyogenic terminal differentiation by *miR-223, miR-195, miR-125* and *miR-146* administration, we search for common shared putative targets. A short list of seven genes (*Wnt5a, Smurf1, Sema5a, Smad3, Foxp1, Fosl2* and *RhoV*) previously involved in myogenesis^95–99^ were assessed, demonstrating that *miR-195* overexpression lead to up-regulation of of all these genes, except *Sema5a*, further supporting their plausible involvement in *miR-195* driven cardiomyogenesis in PE/ST explants. Furthermore, silencing of *Smad3* and *Smurf1* lead to significant down-regulation of early and terminally differentiation markers. Importantly, application of *miR-195* in *siSmad3* and siSmurf1 treated PE/ST explants was unable to rescue the expression of cardiomyogenic lineage markers. Thus, these data demonstrate for the first time that *miR-195* application modulates expression of *Smad3* and *Smurf1*, factors that are essential to promote cardiomyogenesis. It remains unclear whether S*mad3* and *Smurf1* up-regulation by *miR-195* is a direct or an indirect effect. Future experiments will be designed to unravel the molecular mechanisms, although it is important to highlight that microRNAs can directly increase mRNA stability^100^. Overall, these data provide novel insights into the molecular mechanisms whereby *miR-195* administration exerts increased cell differentiation into the cardiomyogenic lineage, i.e. by regulating the expression of *Smad3* and *Smurf1*.

Long non coding RNAs represents a novel emerging class of non coding RNAs with highly diverse cellular functions^101^. Tissue-specific expression of lncRNAs has been widely reported in distinct biological settings, including the cardiovascular system^102^. Seminal studies by Klatenhoff *et al*.^103^ reported the functional role of *Braveheart*, a mesoderm-restricted lncRNA essential for normal lateral plate mesoderm formation and thus cardiac development. Similarly, the function role of handful set of lncRNAs have been reported such as *Fendrr*, *Carmen, Upperhand* and *Tbx5ua*^42,104–106^. However, to date, no lncRNAs has been reported during PE and epicardium formation. We provide herein a systematic analyses of lncRNAs neighboring key growth factors and transcription factors involved in PE and epicardium development and we identify three lncRNAs with enhanced expression in the PE. Secondly, we demonstrate that all three of them and distinctly regulated by cardiomyogenic inductive signals such as thymosin β4^15^ and Bmp^14^ administration as well as by repressive signals, i.e. Fgf signaling. These data support a plausible role for these lncRNAs in PE/ST cardiomyogenic differentiation, however additional experiments are required to dissect their functional role in this context. Regulatory effects of lncRNAs upon microRNAs has been widely reported in the cardiovascular system^107^ as well as in other biological settings^108–113^. However, evidence of microRNA regulation of lncRNAs is still scarce. We provide herein evidences for the first time that microRNAs can modulate the expression of lncRNAs. *miR-195* administration exerts opposite regulatory effects as compared to *miR-23* and *miR-27* supporting a role for these lncRNAs in PE/ST m*iR-195* driven cardiomyogenesis. Surprisingly, *miR-223* did not affect the expression of these lncRNAs, suggesting a microRNA-specific modulation. In sum, our data opened up new pathways to dissect the functional role of microRNAs and lncRNAs in PE/ST development and their plausible application to enhance myocardial formation.

## Supplementary information

Supplementary Table S1.

Supplementary Figure S1.

Supplementary Figure S2.
