# Supplementary material for: MiR-195 enhances cardiomyogenic differentiation of the proepicardium/septum transversum by Smurf1 and Foxp1 modulation
Source: Sci Rep. 2020 Jun 9;10:9334. doi: 10.1038/s41598-020-66325-x (PMC7283354; doi:10.1038/s41598-020-66325-x)
Supplement: Supplementary file 1 — Supplementary Table S1. [file 41598_2020_66325_MOESM1_ESM.pdf]

Genes	Forward (5´>3´)	Reverse (5´>3´)
Gata4	TTCTCAGGCATTCTCGGTCT	GTCCCCATGGTTTTCAGCTA
Mef2c	ACATAATATGCCGCCGTCTG	GGGTGGTGGTACGGTCTCTA
Nkx2.5	TGAAGAGCTACGGGGAGATG	TTGGGCTTGAGAAAGAGGA
Mhy15	ATGCTGAAGAAAGGGCAGAA	GAGCCAGGACCGCACTTA
Tnnt2	CAACATGCTGCACTTTGGAG	AGGTGGTCGATGTTCAGAGG
Wnt5a	AGTACGGATACCGGGAACG	ACCAGAGACACCATGGCACT
Smurf1	CGTGTCCCACTTCAAGGTTT	GCTTTCGGAAGGTTGTCTGT
Sema5a	CCAGTGTCACCAGCTCCTCT	TGCAGTGAGATGTGGATTGAA
Smad3	ATGAGCTTCGTCAAGGGTTG	CCCATCTGTGTGAGGACCTT
Foxp1	AAGGGGCAGTATGGACAGTG	GCAGTAGGTGTGGCTGGTCT
Fosl2	GGATCCGGAGAGAGAGGAAC	CCTTCTGGAGCTCAGCAATC
RhoV	GTCGTCAGCTACACCACCAA	CAGTGTCCCACAGCTGGAT
Slug	CCAGACCCTGGCTACTTCAA	GGAGCAATTTTTGCACTGGT
Snail	CCCTGTGTCTGCAAGATGTG	GAGCAGGTTTTGCACTGGTA
Cdh1	GACCAGGACCAGGACTACGA	GAGATCGGGAACTTCATCGA
Cdh2	GCGGTGGTGAGCAAGACTAT	TCCAGTCAGATCAGCTGCTC
Chd5	GTCCCCATGGTTTTCAGCTA	TGTGAAGAAACCTCCAGGTGA
Col1a1	AAACAGCCGCTTCACCTATG	AGGAGCCAAGTCAATGATGG
Gapdh	TGTCCTCTCTGGCAAAGTCC	TGCCCATTGATCACAAGTTT
Gusb	CGTACCAGCCACTACCCCTA	TTATCCCTGCGGATCAGTTC
siSmurf1	CUGUGAAGAACACGUUGGAUU	UUGACACUUCUUGUGCAACCU
siSmad3	GGUGCUCCAUAUCCUACUAUU	UUCCACGAGGUAUAGGAUGAU
siFosl2	GAGAAGGAGAAACUAGAGUUU	UUCUCUUCCUCUUUGAUCUCA
si Foxp1	CUCCUUAAUAAUAAACCCAUU	UUGAGGAAUUAUUAUUUGGGU
Bmp2_33140	CGTGCTGGGGGGCATGCTGC	GTAACCCTATGGTGTATCA
Bmp2_53839	TATGAACAAGCCAGGCTGAA	CAGTAAGCAGGGTCGGATTC
Wt1_74077	TTGGAGTGCAATGCCAGATA	TCCTTCTCTGCAAAGGTTCTG
Fgf2_56708	GAGACTCTTCCCCACCTCT	TCCTTTTGCTCGAATCTTT
Tcf21_48334	AGGATGGGGAACAGAGTGTG	CCCATGGTTCTTCGTTTTG
Wt1_76127	GAGGAAGGCTGCAGAGGTT	CCAAGAACGTCCTCGGAAA
Bmp4_53170	TCCGTGGCTGTAACTGTTTG	ATGCACCATCTGCTACTGGAA
Fgf8_57126	GAAACAGCAAAAGGCTGCAT	GACACCAAGAGGAGGCTGAG

Supplementary Table 1
