# Supplementary material for: MiR-195 enhances cardiomyogenic differentiation of the proepicardium/septum transversum by Smurf1 and Foxp1 modulation
Source: Sci Rep. 2020 Jun 9;10:9334. doi: 10.1038/s41598-020-66325-x (PMC7283354; doi:10.1038/s41598-020-66325-x)
Supplement: Supplementary file 2 — Supplementary Figure S1. [file 41598_2020_66325_MOESM2_ESM.pdf]

SUPPLEMENTARY FIGURE 1

MiR-195 enhances cardiomyogenic differentiation of the proepicardium/septum transversum by Smurf1 and Foxp1 modulation

Angel Dueñas*, Almudena Expósito*, María del Mar Muñoz, María José de Manuel, Andrea Cámara-Morales, Fabio Serrano-Osorio, Carlos García-Padilla, Francisco Hernández-Torres, Jorge N Domínguez, Amelia Aránega, Diego Franco

Cardiovascular Development Group, Department of Experimental Biology, University of Jaén, Jaén, Spain

*Equal contribution

Corresponding author:

Diego Franco
Cardiovascular Development Group
Department of Experimental Biology, B3-362
University of Jaen, 23071 Jaén
Spain

dfranco@ujaen.es

A

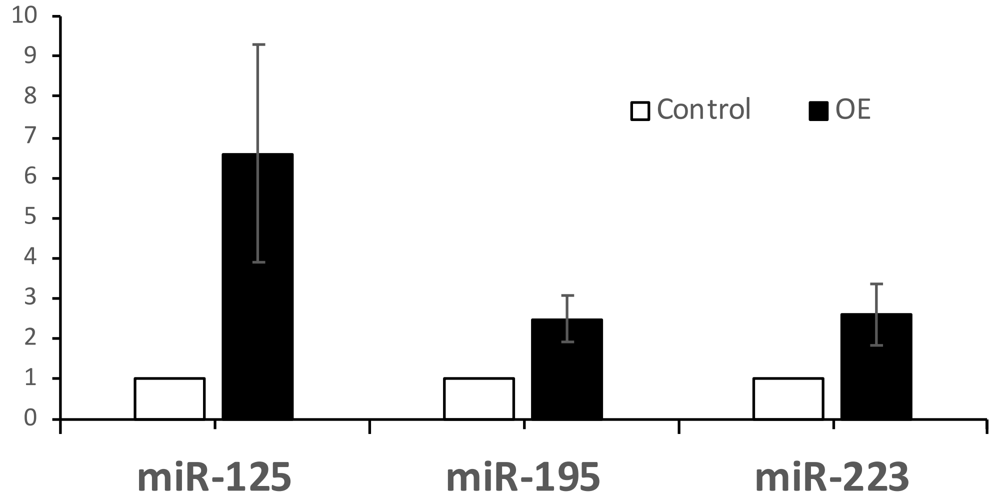

Dueñas et al. Supplementary Figure 1
